# Comparative Fungal Community Analyses Using Metatranscriptomics and Internal Transcribed Spacer Amplicon Sequencing from Norway Spruce

**DOI:** 10.1128/mSystems.00884-20

**Published:** 2021-02-16

**Authors:** Andreas N. Schneider, John Sundh, Görel Sundström, Kerstin Richau, Nicolas Delhomme, Manfred Grabherr, Vaughan Hurry, Nathaniel R. Street

**Affiliations:** a Umeå Plant Science Centre, Department of Plant Physiology, Umeå University, Umeå, Sweden; b Department of Biochemistry and Biophysics, National Bioinformatics Infrastructure Sweden, Science for Life Laboratory, Stockholm University, Solna, Sweden; c Umeå Plant Science Centre, Department of Forest Genetics and Plant Physiology, Swedish Agricultural University, Umeå, Sweden; d Department of Medical Biochemistry and Microbiology, Uppsala University, Uppsala, Sweden; Pacific Northwest National Laboratory

**Keywords:** fungi, metatranscriptomics, ITS amplicon sequencing, Norway spruce, nutrient enrichment, ectomycorrhiza, tree roots, phyllosphere, fungi, phyllosphere-inhabiting microbes

## Abstract

The health, growth, and fitness of boreal forest trees are impacted and improved by their associated microbiomes. Microbial gene expression and functional activity can be assayed with RNA sequencing (RNA-Seq) data from host samples. In contrast, phylogenetic marker gene amplicon sequencing data are used to assess taxonomic composition and community structure of the microbiome. Few studies have considered how much of this structural and taxonomic information is included in transcriptomic data from matched samples. Here, we described fungal communities using both host-derived RNA-Seq and fungal ITS1 DNA amplicon sequencing to compare the outcomes between the methods. We used a panel of root and needle samples from the coniferous tree species *Picea abies* (Norway spruce) growing in untreated (nutrient-deficient) and nutrient-enriched plots at the Flakaliden forest research site in boreal northern Sweden. We show that the relationship between samples and alpha and beta diversity indicated by the fungal transcriptome is in agreement with that generated by the ITS data, while also identifying a lack of taxonomic overlap due to limitations imposed by current database coverage. Furthermore, we demonstrate how metatranscriptomics data additionally provide biologically informative functional insights. At the community level, there were changes in starch and sucrose metabolism, biosynthesis of amino acids, and pentose and glucuronate interconversions, while processing of organic macromolecules, including aromatic and heterocyclic compounds, was enriched in transcripts assigned to the genus *Cortinarius*.

**IMPORTANCE** A deeper understanding of microbial communities associated with plants is revealing their importance for plant health and productivity. RNA extracted from plant field samples represents the host and other organisms present. Typically, gene expression studies focus on the plant component or, in a limited number of studies, expression in one or more associated organisms. However, metatranscriptomic data are rarely used for taxonomic profiling, which is currently performed using amplicon approaches. We created an assembly-based, reproducible, and hardware-agnostic workflow to taxonomically and functionally annotate fungal RNA-Seq data obtained from Norway spruce roots, which we compared to matching ITS amplicon sequencing data. While we identified some limitations and caveats, we show that functional, taxonomic, and compositional insights can all be obtained from RNA-Seq data. These findings highlight the potential of metatranscriptomics to advance our understanding of interaction, response, and effect between host plants and their associated microbial communities.

## INTRODUCTION

A growing body of research shows that plants harbor a complex assemblage of epiphytic and endophytic symbionts (1). Understanding the composition and role of the microbial components of such systems raises fundamental questions concerning the taxonomic composition and biological functions provided by these communities and how they influence plant survival and fitness. High-throughput DNA sequencing technologies have vastly improved our ability to assay these complex and diverse microbial communities (2, 3). The current *de facto* standard of metagenomics is the use of amplicons spanning regions of marker genes, usually the internal transcribed spacer (ITS) region of the rRNA gene for fungal species and variable regions of the 16S rRNA gene for bacteria (4, 5), for both of which extensive reference databases exist (6, 7). Although useful for taxonomic profiling, DNA-based amplicon methods suffer from methodological biases such as not accounting for multiple rRNA copies per cell and preferential primer binding, leading to bias for or against certain taxa (8, 9). Moreover, DNA-based methods cannot differentiate between living and dead sources of DNA (10). In contrast to examining DNA, RNA sequencing (RNA-Seq) captures actively expressed sequences as well as their relative abundance. Among the numerous appealing qualities of RNA-Seq is the nearly universal coverage of the transcriptome. In the current context, and particularly in the case of field samples, that coverage comprises transcripts from both a host and its associated microbial community, enabling what we previously referred to as serendipitous metatranscriptomics (11) and what others have termed tripartite sequencing (12) or, in the case of a host and single microbial species, dual RNA-Seq (13). One characteristic of using metatranscriptome (i.e., the transcriptome of a whole community) data is that it yields insight into the biological processes active within microbial communities, providing functional insights (14–18). The availability of functional information from both components of the holobiont system (the assemblage of the plant host and the hosted microbial community) could be transformative in advancing our understanding of the development, dynamics, interactions, and effects of these two components (17, 19, 20). In principle, RNA-Seq applied to a holobiont system would enable taxonomic profiling of the represented species, offering taxonomic information in addition to information on the biological processes actively represented in the metatranscriptome.

There have been few systematic comparisons of metatranscriptomics data to those from amplicon sequencing of the 16S or ITS regions of rRNA genes. One study using human stool samples concluded that total metatranscriptome data have higher sensitivity and reproducibility than both ITS and 16S amplicon data (21). More such studies are needed to understand whether both methods provide similar insight into community diversity, species composition, and biological function. Here, this question was addressed by performing a comparison of taxonomic information and community structure obtained from mRNA-based metatranscriptomics and amplicon sequencing of the fungal ITS1 region. As a study system, we used a panel of root and needle samples from *Picea abies* (Norway spruce) growing in northern boreal Sweden. The boreal forest covers around one-third of the world’s forested areas and is mostly characterized by harsh climates and N-limited plant growth (22). These forests are dominated by conifers, and they host complex communities of microorganisms, both in the soil and in close association with the forest trees. Ectomycorrhizal (ECM) fungi are especially important in this context, colonizing over 90% of root tips in the boreal forest (23). ECM enhance tree nutrient uptake and are important drivers of carbon and nutrient cycling in the boreal forests (24). Another ubiquitous and important group of fungi are saprotrophs, which play an important role in degrading organic litter and root detritus (25). The site sampled in this study is part of a controlled short-term (5 years) and long-term (25 years) nutrient enrichment (NE) experiment including untreated nutrient-deficient (ND) control plots (26, 27). The aim of this study was 3-fold: (i) to implement a bioinformatic workflow for metatranscriptomic RNA-Seq data that filters host-derived reads and assigns taxonomic and functional annotations to assembled fungal transcripts; (ii) to compare results derived using this pipeline to rRNA gene amplicon-based data of the fungal ITS region (28); and (iii) to demonstrate the potential of our metatranscriptomic data for providing multifaceted, functional insight into actively expressed genes, for example, identifying biological processes that are enriched in response to long-term NE both at community level and for the selected genus *Cortinarius*.

## RESULTS

### Pipeline development and data set statistics.

**FIG 1:**
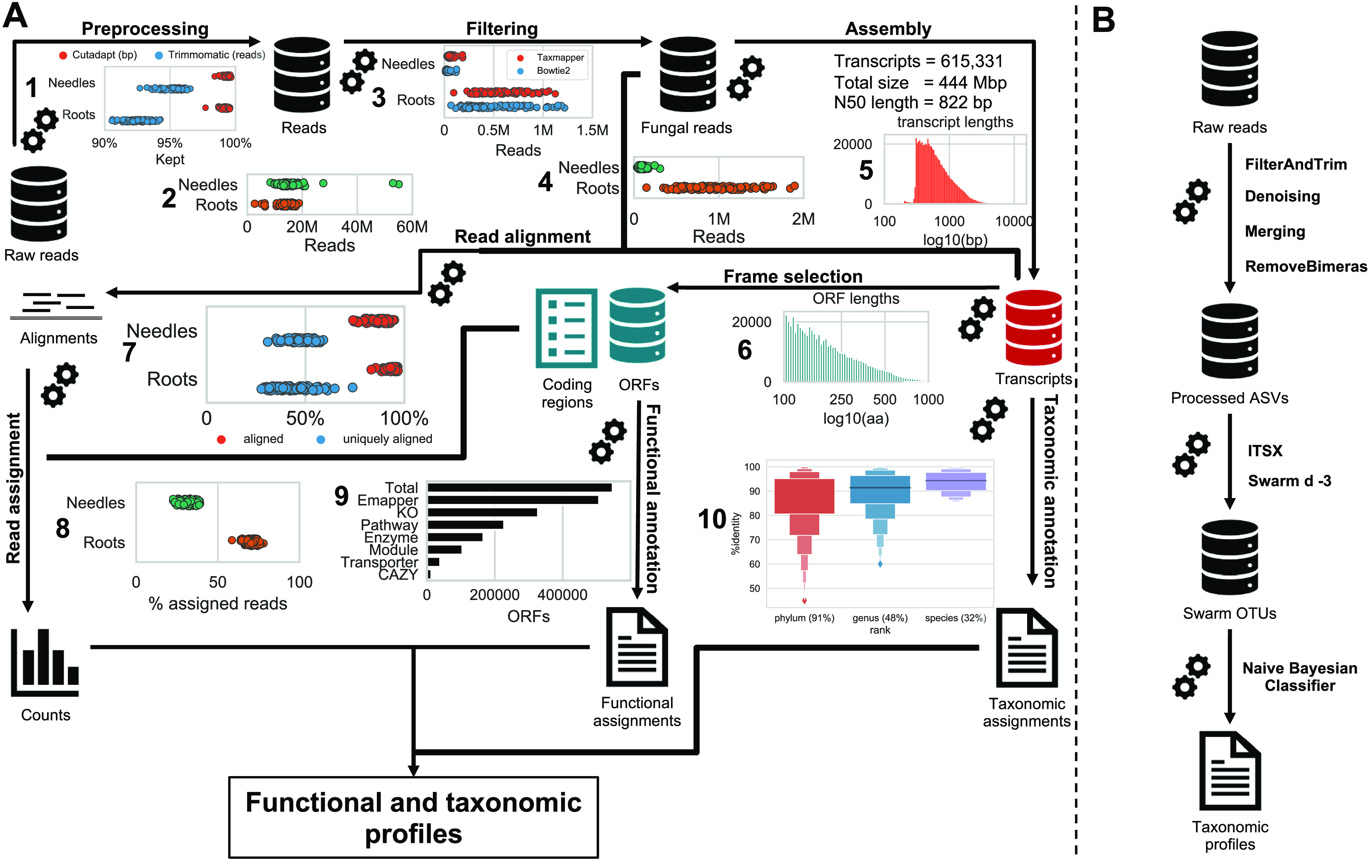
(A) Overview of the RNA-Seq workflow. (Step 1) The inset shows the proportion of the raw data that was kept after trimming adapters and removing low-quality regions (shown in base pairs for cutadapt and as reads for Trimmomatic). (Step 2) Reads remaining after preprocessing in needle and root samples. (Step 3) The inset shows the number of reads identified as fungal by bowtie2 alignments and TaxMapper assignments in needle and root samples. (Step 4) Number of fungal reads in needle and root samples after filtering. (Step 5) The inset shows length distribution of assembled transcripts (log_10_ scale) after assembly using Megahit. (Step 6) Length distribution of the open reading frames (ORFs) as determined by GeneMarkS-T (in amino acids, log_10_ scale). (Step 7) The inset shows overall and uniquely aligned fraction of reads for needle and root samples, after aligning fungal reads to the assembled transcripts using bowtie2. (Step 8) Fraction of assigned reads in needle and root samples, as determined by FeatureCounts. (Step 9) The inset shows number of total ORFs and number of ORFs with different levels of functional annotations obtained from eggnog-mapper. (Step 10) Distribution of amino acid identity for DIAMOND BLASTX hits used to assign taxonomy at the different ranks, using the contigtax tool. The fraction of transcripts assigned at each rank is shown in parentheses on the *x* axis. Full statistics on surviving reads are available in Table S1. (B) Overview of the amplicon sequencing workflow. Raw, demultiplexed reads were filtered and trimmed, after which dada2 was used to denoise the reads and to merge forward and reverse reads. Subsequently, chimeras were removed, and the resulting amplicon sequencing variants (ASVs) were cut with ITSx and clustered into Swarm operational taxonomic units (SOTUs). Finally, taxonomy was assigned to SOTUs using the dada2 naive Bayesian classifier and the UNITE database. Detailed read counts can be found in Table S1.

RNA-Seq of roots and needles yielded an average 14.7 million reads per sample after adapter/quality trimming (Fig. 1A), of which 0.6% (89,763 reads) and 6.7% (933,229 reads) on average were identified as fungal (by alignment to the JGI MycoCosm and TaxMapper databases) in the needle and root samples, respectively (see Table S1 in the supplemental material). Assembly of fungal reads using Megahit generated 615,331 transcripts, with a total size of 444 Mbp. The length of transcripts ranged from 200 to 12,588, with an *N*_50_ length of 822 bp (for an Ex*N*_50_ [i.e., the *N*_50_ value over the most highly expressed genes that represent *x*% of the total normalized expression data] graph and a comparison to Trans-ABySS and Trinity, see Fig. S1 and Text S1). A total of 547,305 open reading frames (ORFs) were called on the assembled transcripts, with a median length of 98 amino acids, while for 68,029 (11.1%) transcripts no ORF was found. An average of 34.3% reads in needle samples and 70.6% in root samples were aligned to these ORFs. Functional annotation of ORFs was performed, with 92.7% of ORFs having a hit in the eggnog database, of which 64.5% were assigned to a Kyoto Encyclopedia of Genes and Genomes (KEGG) ortholog and 59.8% were assigned Gene Ontology (GO) terms. Taxonomic assignments resulted in 95.5%, 50.4%, and 34.2% of transcripts assigned at the phylum, genus, and species levels, respectively. A more detailed description is available in Text S1.

10.1128/mSystems.00884-20.1 TEXT S1 Supplemental methods, including an extended description of methods, with all parameters and version of tools and programs used. Supplemental results and discussion, including phyllospheric results and more detailed random forest analyses. Download Text S1, DOCX file, 0.05 MB. Copyright © 2021 Schneider et al. 2021 Schneider et al. https://creativecommons.org/licenses/by/4.0/ This content is distributed under the terms of the Creative Commons Attribution 4.0 International license.

10.1128/mSystems.00884-20.2 FIG S1 Length and read assignment statistics of metatranscriptomic assemblies using megaHIT, Trans-ABySS, and Trinity. (A) (Top row, from left to right, all colored by assembler software) Number of assembled transcripts per assembly; total size per assembly in base pairs; *N*_50_ length per assembly. (Bottom row) Assembly size in base pairs plotted over transcript length in base pairs; overall alignment rate as reported with bowtie2, when mapping reads to assembled transcripts. Each point is one sample; Percentage of reads (of total preprocessed reads) assigned to open reading frames (ORFs) called on transcripts using featureCounts. Only reads with a mapping quality score (mapQ) of ≥10 were considered by featureCounts. (B) Graph showing the Ex*N*_50_ of the megaHit transcript assembly (i.e., the *N*_50_ value over the most highly expressed genes that represent *x*% of the total normalized expression data). Download FIG S1, EPS file, 1.4 MB. Copyright © 2021 Schneider et al. 2021 Schneider et al. https://creativecommons.org/licenses/by/4.0/ This content is distributed under the terms of the Creative Commons Attribution 4.0 International license.

10.1128/mSystems.00884-20.8 TABLE S1 Sample metadata and read survival statistics. (1) Metadata of all samples, including European nucleotide archive (ENA) IDs. (2) RNA data read counts through pipeline steps. (3) ITS amplicon data read counts through pipeline steps. Download Table S1, XLSX file, 0.06 MB. Copyright © 2021 Schneider et al. 2021 Schneider et al. https://creativecommons.org/licenses/by/4.0/ This content is distributed under the terms of the Creative Commons Attribution 4.0 International license.

For the ITS1 amplicon sequencing data (Fig. 1B), between 86,279 and 338,800 reads per sample remained (176,734 on average), corresponding to a range between 47 and 78% of the raw reads (Table S1). Denoising and chimera removal resulted in 5,726 ASVs in total, of which 2,694 were found in roots and 3,032 in needle samples. After clustering, there were 2,673 Swarm operational taxonomic units (SOTUs), 1,172 in root samples and 1,890 in needle samples.

### Comparison of tree tissues in nutrient deficient control samples.

**FIG 2:**
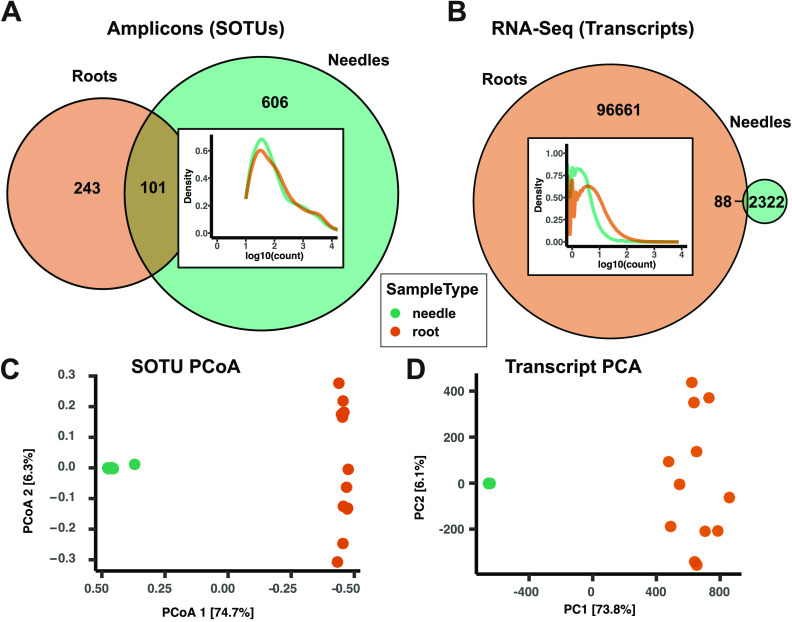
Nutrient deficient (ND) sample overview, contrasting fungal communities in Norway spruce needles and roots. (A and B) Venn diagrams showing the number of postfiltering fungal Swarm operational taxonomic units (SOTUs) (A) and fungal transcripts (B) obtained from root and needle ND samples. Inside the Venn diagrams are density curves showing the log_10_-transformed total count distribution in needle (green) and root (brown) samples. (C) Principal-coordinate analysis (PCoA) of Bray-Curtis dissimilarities between needle and root ND samples, obtained from ITS1 amplicon sequencing data. (D) Principal-component analysis (PCA) of variance stabilization-transformed counts from the metatranscriptomes of Norway spruce roots and needles.

Twice as many SOTUs were observed in the needle ND samples as in roots (Fig. 2A), consistent with the published analysis of these data (28). In contrast, the RNA-Seq data set showed around 50 times more remaining transcripts after abundance filtering in the root than in the needle samples (Fig. 2B). There was a predominance of low counts per SOTU and transcript in the ITS and the RNA data sets, respectively, particularly in the needle samples (Fig. 2A and B, insets).

A principal-coordinate analysis on the Bray-Curtis dissimilarities between root and needle ND samples in the ITS data set revealed a clear separation of needle and root samples along the first principal coordinate (75% explained variance) (Fig. 2C). The second principal coordinate was characterized by the biggest variation among the root samples, corresponding to the variation between field plots. A principal-component analysis of transcript counts of root and needle ND samples led to a highly similar separation and arrangement of samples (Fig. 2D), but with lower variation among needle samples. The visual congruency between the two data sets for the ND samples was confirmed (Mantel *r*, 0.89; *P* < 0.001; Procrustes correlation, 0.86; squared m12, 0.26; *P* < 0.001). Due to the small amount of remaining transcripts and the low intersample variance in the needle samples, only root samples were used for later data comparisons.

### Comparison of taxonomic annotations in ITS and RNA databases and data sets.

**FIG 3:**
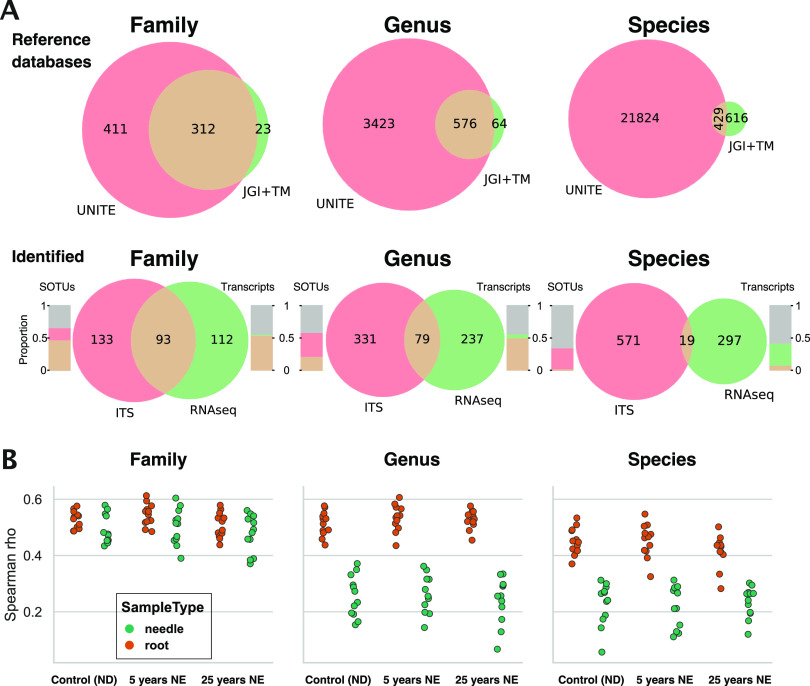
Taxonomic congruence between RNA-Seq and ITS amplicon sequencing data sets. (A) (Top) Venn diagrams showing taxonomic unit overlap between the UNITE database and the JGI MycoCosm and TaxMapper (JGI+TM) genome databases at the family, genus, and species levels. (Bottom) Taxonomic unit overlap of identified taxa in the RNA and the ITS sequencing data sets. Bars indicate the proportion of transcripts and Swarm operational taxonomic units (SOTUs) belonging to the unique and common portions of the Venn diagrams (colors correspond; gray indicates unidentified transcripts/SOTUs on the corresponding taxonomic level). (B) Spearman rank correlations of taxonomic abundance (family, genus, and species levels) between RNA-Seq and ITS amplicon sequencing samples. Colors indicate samples of root (brown) or needle (green) origin.

To compare the coverage of the databases used for taxonomic annotation of transcripts (JGI MycoCosm and TaxMapper) and SOTUs (UNITE database), the number of families, genera, and species listed in both or only one of the databases was assessed (Fig. 3A). The proportional overlap between the two databases clearly decreased with lower taxonomic levels. A similar trend was found for taxa identified in the two data sets, but with a lower proportional overlap at the species level than between the databases (Fig. 3A, lower row). At the family level, the percentage of common transcripts and SOTUs was ∼50%, while at the species level, <5% were in common (Fig. 3A, area bar graphs). The same trend was apparent on a read count level (Fig. S2A).

10.1128/mSystems.00884-20.3 FIG S2 Percentages and correlations of taxonomic overlap between the ITS and the RNA data set. (A) (Left) Bar graphs visualizing proportions of transcripts and SOTUs assigned to taxonomic units shared between the RNA and ITS data sets (yellow) or unique to the respective data sets (green/red). Grey signifies no taxonomy assigned at the respective level. (Right) Bar graphs with same color coding but with percentages based on the number of reads assigned to shared and unique taxonomic units. Data are separated into needle (left) and root (right) samples. (B) Spearman rank correlations of taxonomic abundance (phylum, class, and order levels) between RNA-Seq and ITS amplicon sequencing samples. Colors indicate samples of root (brown) or needle (green) origin. Download FIG S2, EPS file, 1.0 MB. Copyright © 2021 Schneider et al. 2021 Schneider et al. https://creativecommons.org/licenses/by/4.0/ This content is distributed under the terms of the Creative Commons Attribution 4.0 International license.

To assess how well the relative abundance of common taxa agreed between the RNA and the ITS data sets, Spearman rank correlations were computed for all taxonomic levels (Fig. 3B; Fig. S2B). At the family level, the correlations ranged between 0.4 and 0.6 (medians, 0.53 in roots and 0.48 in needles). In accordance with the Venn diagrams, correlations decreased rapidly at lower taxonomic ranks in needle samples (medians, 0.48 and 0.26 at the genus and species levels, respectively) and moderately in root samples (medians, 0.53 and 0.44 at the genus and species levels, respectively).

### Comparison of community structure in root samples.

**FIG 4:**
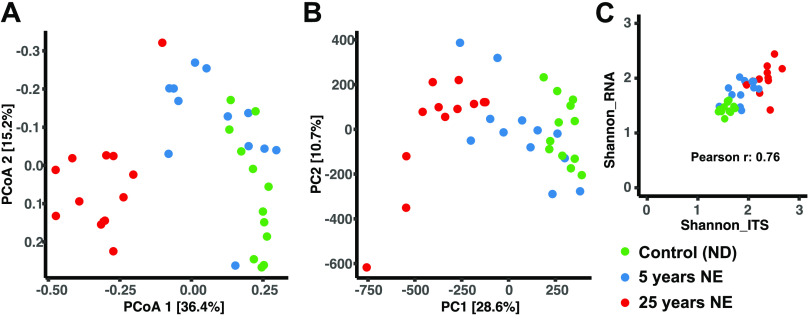
Ordination and alpha diversity index comparison of root samples. (A) Principal-coordinate analysis of rarefied Swarm operational taxonomic unit (SOTU) counts, colored by treatment. (B) Principal-component analysis of variance stabilization transformed transcript counts, colored by treatment. (C) Sample-wise relationship between Shannon diversity index values (genus level) using ITS amplicon sequencing (*x* axis) and RNA-Seq (*y* axis), colored by treatment.

To assess how well the transcript and SOTU data from the root samples structurally correlated with each other, within and between fertilization treatments, ordination analyses were performed. A principal-coordinate analysis (PCoA) on the SOTU counts obtained from the ITS1 sequencing data revealed the same pattern as in the previously published results (Fig. 4A) (28). The 25-year-treated (NE-25) samples clearly separated from the controls on the first principal coordinate (explaining 36% of the variance). A permutational multivariate analysis of variance (PERMANOVA) test showed significance for fertilization treatment (*P* < 0.001) but not for sampling date. Similarly, a principal-component analysis (PCA) of the transcript counts (Fig. 4B) showed that the NE treatment accounted for the highest variance in the data set (29%) and that after 25 years of NE, the fungal transcriptomes in the fertilized plots were distinct from those in the ND samples. PERMANOVA confirmed this (*P* < 0.001), while sampling date was not significant. The correlation between the sample distances in the two ordinations was significant (Mantel *r*, 0.74; *P* < 0.001; Procrustes correlation, 0.55; squared m12, 0.69; *P* < 0.001). Phyllospheric community structure comparisons (Fig. S3) are discussed in the supplemental material (Text S1).

10.1128/mSystems.00884-20.4 FIG S3 Ordinations and diversity index comparison of needle samples. (A) Principal-coordinate analysis of rarefied Swarm operational taxonomic unit (SOTU) counts, colored by seasonal time point. (B) Principal-component analysis of transcript counts, colored by time point. (C) Sample-wise relationship between Shannon diversity index values (genus level) using ITS amplicon sequencing (*x* axis) and RNA-Seq (*y* axis), colored by time point. Download FIG S3, EPS file, 0.3 MB. Copyright © 2021 Schneider et al. 2021 Schneider et al. https://creativecommons.org/licenses/by/4.0/ This content is distributed under the terms of the Creative Commons Attribution 4.0 International license.

Furthermore, Shannon diversity index values at the genus level between the two data sets were compared (Fig. 4C). The total correlation was strong (Spearman *r*, 0.76), and the increase in Shannon diversity with longer NE treatment (as reported in reference 28) was apparent in both the ITS and the RNA data (all pairwise comparisons were significant; all *P* < 0.02).

### Comparison of highly abundant families and random forest classification by sample source using taxonomic annotations in both data sets.

**FIG 5:**
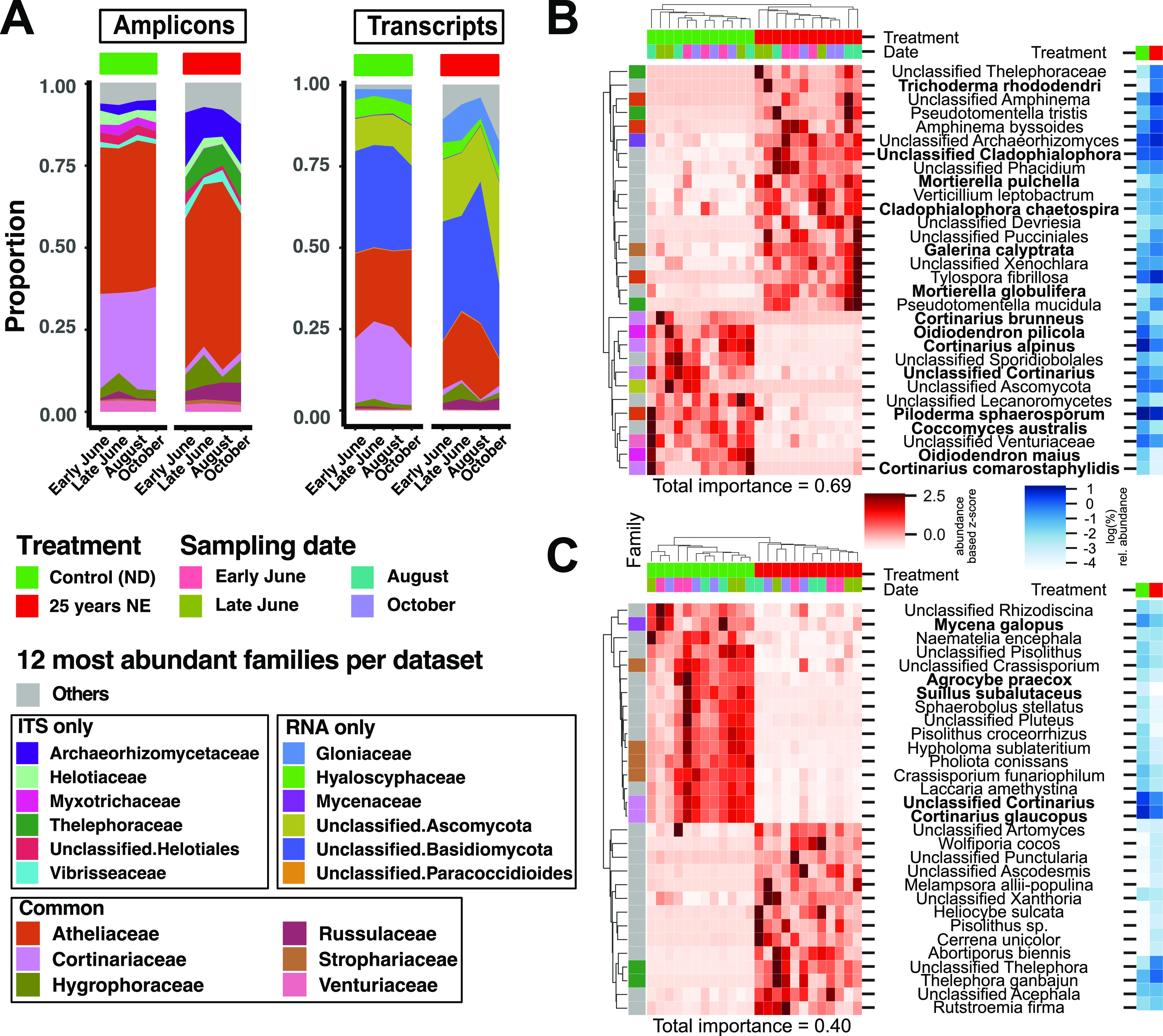
Taxonomic overview and random forest results. (A) Taxonomic overview area plot of ITS amplicon sequencing data (left bars) and annotated transcripts (right bars) from root samples. Counts were summed to the family level, and mean relative abundance per sample type is displayed. Shown in color inside the bars are the 12 most abundant families. Red and green bars on top indicate the treatment; sampling time point is indicated below the columns. (B) ITS data random forest results. Heat map showing the distribution of the 30 most important species (rows) for prediction of samples (columns) into ND/25-year NE groups. Normalized abundance values (summed to the species level) were converted to z-scores per row to highlight differences between samples. Hierarchical clustering of samples and species was performed using correlation metrics and complete linkage clustering. Colors on top indicate treatment and sampling date. Colors in the left margin indicate the corresponding family (gray indicates that the result was not among the 12 most abundant family annotations in any of the two data sets). The two-column heat map to the right indicates average relative abundance in ND and NE samples. Species in bold belong to genera that are found in both data sets. (C) Metatranscriptome random forest results. Heat map showing the distribution of the top 30 most important species (rows) for prediction of samples (columns) into control/25-year-treatment groups. Normalized expression values (summed to species level) were converted to z-scores per row to highlight differences between samples. Hierarchical clustering of samples and species was performed using correlation metrics and complete linkage clustering. Colors on top indicate treatment and sampling date. Colors in the left margin indicate the corresponding family of species. The two-column heat map to the right indicates average relative abundance in control and NE samples. Species in bold belong to genera that are found in both data sets.

Counts in both data sets were summarized to the family level, and relative proportions of the 12 most abundant family annotations from the two data sets were visualized (Fig. 5A). As expected from the above comparison of taxonomic annotations, the general overlap was not strong, but notable examples, like Cortinariaceae and Hygrophoraceae, showed very similar abundance distributions. The total proportion occupied by the 12 most abundant family annotations (covering 95% of reads on average) in each data set were highly similar, and this proportion decreased with longer NE treatment.

A random forest classifier on both ITS and RNA data set was used to classify samples by treatment and date. The 30 species having the highest importance in both data sets were then compared (Fig. 5B and C). For a more detailed description of the random forest results, see Text S1, Fig. S4 and S5, and Table S2. When classification of root samples by treatment type (ND, NE-5, and NE-25) was compared, predictive accuracy was high (>0.7) in both the ITS and RNA data. When only ND and NE-25 samples were used, the accuracy increased to 1 in both data sets. Root samples could not be accurately classified by sampling date, congruent with the earlier ordination-based statistical tests. In the ITS data, the 30 most important species had a summed importance of 0.69 and 17 species fell among the 12 most abundant families in the ITS data set (Fig. 5A). In addition, the mean relative abundance of a species and its feature importance had a Spearman rank correlation coefficient of 0.79. For the averaged RNA data, the top 30 most important species had summed importance of 0.4 (Fig. 5C), and in contrast to the ITS data set, only 9 of the top 30 important species belonged to the most abundant families (Fig. 5B); the Spearman correlation coefficient for mean relative abundance and importance was only 0.13.

10.1128/mSystems.00884-20.5 FIG S4 Random forest (RF) accuracy statistics summary. Violin plots showing distribution of random forest classification accuracies per sample. Average numbers can be found in Table S2. (A) (Left) Classification of all treatments (ND, NE-5, NE-25), in root samples (top) and needle samples (bottom). (Right) Classification accuracies using long term NE and ND (ND, NE-25) samples only. All panels are color coded by dataset (red, RNA; blue, ITS). (B) RF classification accuracies using sample date (Early_June, Late_June, August, October) as the classification category. All panels are color coded by data set (red, RNA; blue, ITS). (C) Comparison of classification accuracies of root and needle samples without summarizing technical replicates and without an abundance filter threshold (red, RNA_f) to the summed and filtered RNA data set (blue, RNA). The classification categories are the same as in panel A. Download FIG S4, EPS file, 2.0 MB. Copyright © 2021 Schneider et al. 2021 Schneider et al. https://creativecommons.org/licenses/by/4.0/ This content is distributed under the terms of the Creative Commons Attribution 4.0 International license.

10.1128/mSystems.00884-20.6 FIG S5 Metatranscriptome random forest results without merging technical replicates and without abundance filtering. Heat map showing the distribution of the top 30 most important species (rows) for prediction of samples (columns) into control/25-year-treatment groups. Normalized expression values (summed to species level) were converted to z-scores per row to highlight differences between samples. Hierarchical clustering of samples and species was performed using correlation metrics and complete linkage clustering. Colors on top indicate treatment and sampling date. Colors in the left margin indicate the corresponding family (grey indicates that the result is not among the 12 most abundant family annotations in either of the data sets) of species. Download FIG S5, EPS file, 2.3 MB. Copyright © 2021 Schneider et al. 2021 Schneider et al. https://creativecommons.org/licenses/by/4.0/ This content is distributed under the terms of the Creative Commons Attribution 4.0 International license.

10.1128/mSystems.00884-20.9 TABLE S2 Random forest classification accuracy values. (1) All classification accuracy mean values for random forest analyses of RNA and ITS data. (2) Random forest classification accuracies on technical replicates, not summarized and without abundance filtering applied. Download Table S2, XLSX file, 0.01 MB. Copyright © 2021 Schneider et al. 2021 Schneider et al. https://creativecommons.org/licenses/by/4.0/ This content is distributed under the terms of the Creative Commons Attribution 4.0 International license.

### Functional annotation of RNA data provides functional insight into fungal-community activity.

**FIG 6:**
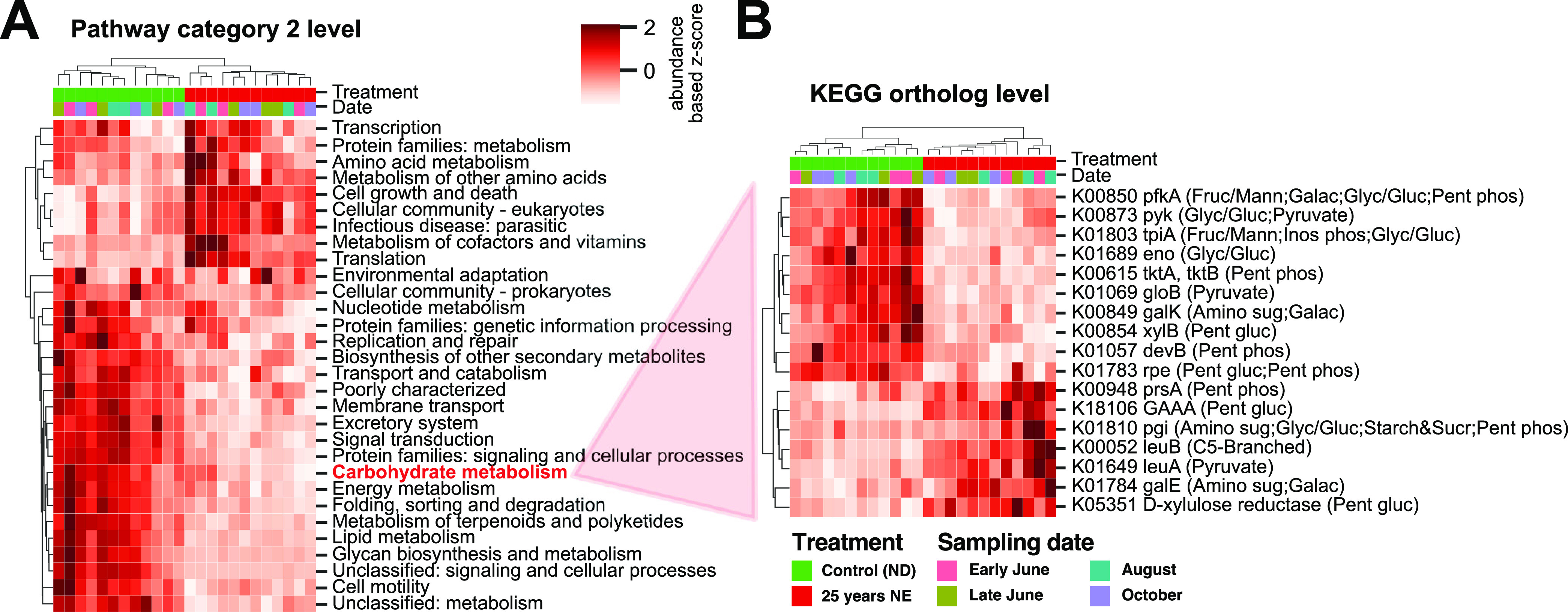
Functional analysis—random forest results. (A) Heat map of the 50% KEGG orthologs with the highest importance, summed to pathway category 2 level. Normalized expression values are converted to z-scores per row to highlight differences between samples. Hierarchical clustering of samples and KEGG pathways was performed using correlation metrics and complete linkage clustering. Colors on top indicate treatment and sampling date. (B) KEGG orthologs falling into the “Carbohydrate metabolism” pathway category. Visualization was performed as described for panel A.

As described above for taxonomic assignment, random forest analysis was applied using functional expression profiles of fungal transcripts to classify samples. Normalized expression values for ORFs assigned to the kingdom Fungi were summed to the KEGG ortholog (KO) level. To gain insight as to which functional categories were important for separating ND and NE-25 samples, the expression of KOs with a combined importance of 0.5 was summed to higher level functional categories in the KEGG pathway hierarchy (Fig. 6A). This revealed that the transcription, translation, and amino acid metabolism categories had higher expression values in NE-25 samples, while, e.g., carbohydrate metabolism, nucleotide and lipid metabolism, and signal transduction categories were more highly expressed in control samples.

Evidence in the literature suggests that N addition leads to a decrease in tree belowground carbon allocation to ECM (29, 30), accompanied by a decrease in ectomycorrhizal growth (31). The random forest results were therefore used to explore the “carbohydrate metabolism” KEGG pathway category. In total, 17 KOs from this pathway category were among the most important 50% of KOs (Fig. 6B). Noteworthy orthologs with decreased abundance after long-term NE included several key players in the major glucose conversion pathway glycolysis (6-phosphofructokinase, pyruvate kinase, and triosephosphate isomerase), the glycolysis-parallel pentose phosphate pathway (transketolase and 6-phosphogluconolactonase), and others from closely related downstream pathways. Some of the higher-abundance KOs were related to amino acid (specifically, leucine and isoleucine) metabolism: 3-isopropylamate dehydrogenase and synthase.

### Long-term nutrient enrichment leads to major functional changes at the community level with seasonal differences.

**FIG 7:**
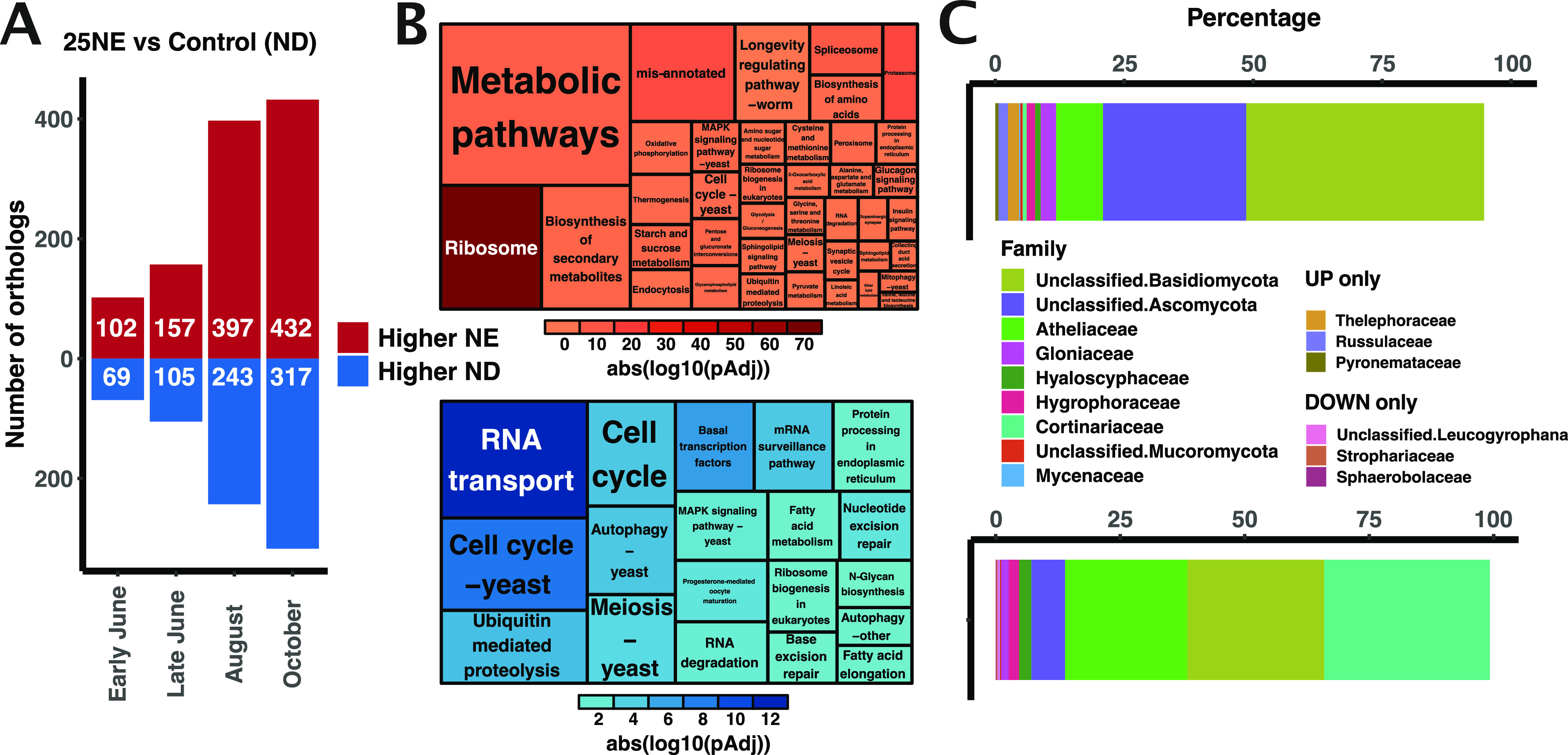
Functionally summarized differential KEGG ortholog (KO) abundance overview. (A) Bar plot showing the number of KOs found to be significantly more (red) or less (blue) abundant after 25 years of nutrient enrichment (NE) in comparison to the untreated controls. (B) KEGG pathway enrichment of all KOs identified as significantly differentially abundant at all four sampling dates, after 25 years of NE. Color intensity is determined by adjusted *P* value of enrichment, while rectangle size is proportional to the number of differentially abundant KOs in the respective pathway category. “Mis-annotated” refers to pathway categories associated with human diseases. Table S3 contains all terms and statistics. (C) Taxonomic distribution at the family level of all transcripts assigned to the set of KOs displayed in panel A. Only the 12 most abundant family annotations from both sets of KOs are displayed.

A differential abundance analysis of the 25 years versus control condition identified KOs (1,822 KOs overall and 1,189 unique KOs), with an increasing number of differentially abundant KOs throughout the growing season (Fig. 7A). There were 47 commonly differentially abundant KOs at all four sampling dates (6 to 27% of KOs per seasonal time point), of which 29 increased and 18 decreased in abundance.

**FIG 8:**
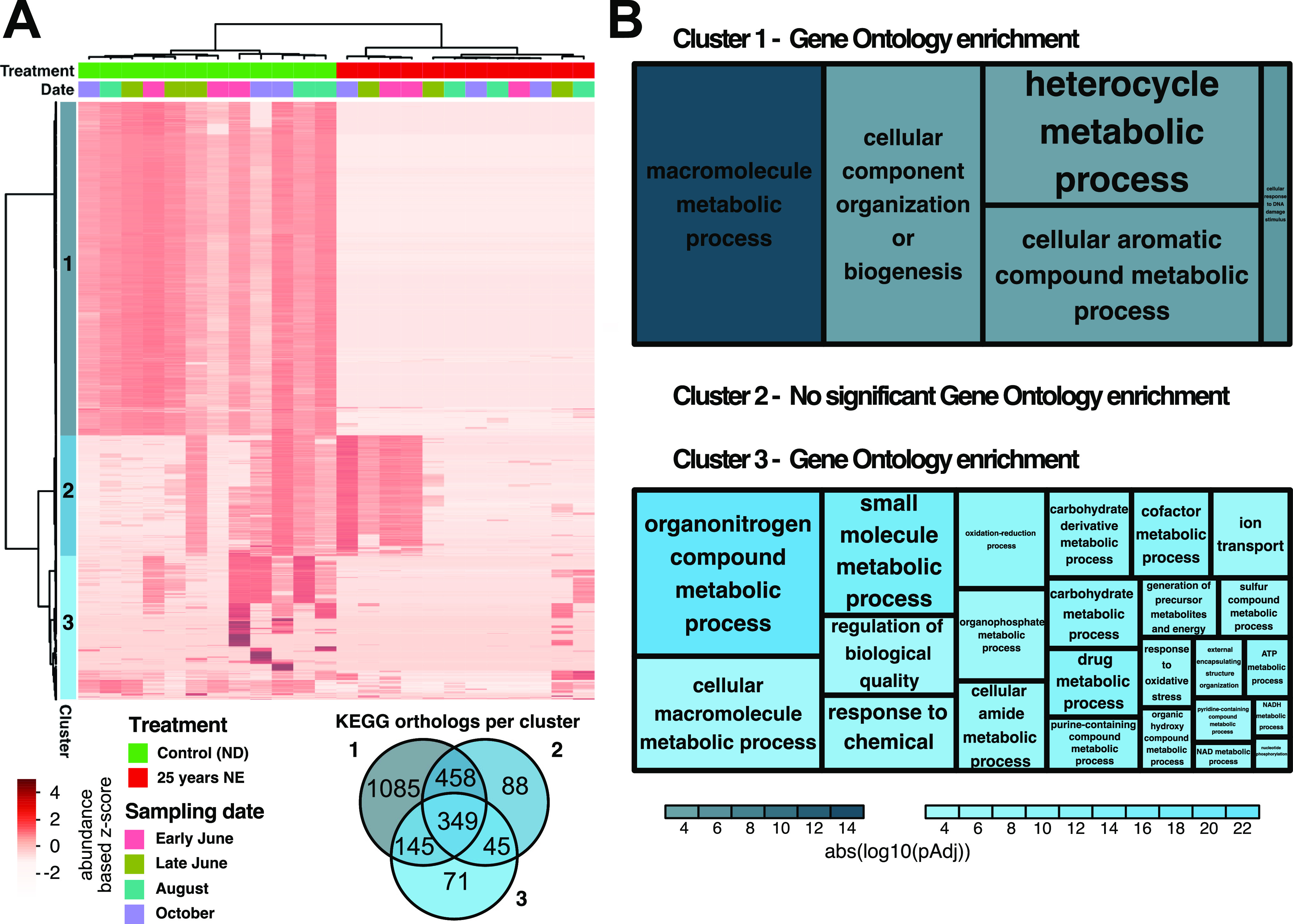
Hierarchical clustering of transcripts assigned to the genus *Cortinarius* and Gene Ontology (GO) enrichment. (A) Heat map of all transcripts assigned to the genus *Cortinarius*. Normalized variance stabilization transformed expression values were transformed to z-scores per row to highlight differences between samples. Hierarchical clustering of samples and transcripts was performed using Ward’s minimum variance method. Colors on top indicate treatment and sampling date, while colors to the left indicate the three highest-level clusters. A color legend is provided below the heat map. The Venn diagram below the heat map shows KEGG orthologs (KOs) derived from the three clusters and the respective overlap between them. (B) Tree maps showing GO enrichment of all transcripts identified as belonging to the three highest level clusters. The upper tree map summarizes significant GO enrichments for cluster 1 (4,047 transcripts), and the lower tree map shows the enrichment for cluster 3 (1,741 transcripts). There were no significant enrichments for cluster 2 (1,465 transcripts). Color intensity is determined by adjusted *P* value of the enrichment, while rectangle size is proportional to the number of transcripts mapping to the respective GO term. Table S3 shows all terms and corresponding statistics.

10.1128/mSystems.00884-20.10 TABLE S3 Enriched differentially abundant KEGG ortholog (KO) and Gene Ontology (GO) details. (1) Full table of enriched differentially abundant KEGG orthologs (differentially abundant KOs) displayed as a tree map in Fig. 7B, including statistics. (2) Full table of enriched GO terms displayed as treemap in Fig. 8B, including statistics. Download Table S3, XLSX file, 0.02 MB. Copyright © 2021 Schneider et al. 2021 Schneider et al. https://creativecommons.org/licenses/by/4.0/ This content is distributed under the terms of the Creative Commons Attribution 4.0 International license.

KEGG pathway enrichment of differentially abundant KOs (Fig. 7B) showed that general housekeeping pathways, including ribosome, proteasome, and spliceosome, were enriched among more highly abundant KOs, pointing to major shifts in biological activity. Pathways such as RNA transport, cell cycle, ubiquitin-mediated proteolysis, and mRNA surveillance were enriched among KOs with lower abundance. These enrichments were similar to results from the random forest analysis (Fig. 6). Notable and more specific pathways composed of KOs with higher abundance included starch and sucrose metabolism, biosynthesis of amino acids, and pentose and glucuronate interconversions. Specific pathways that were found to have significantly lower abundance after NE included autophagy, fatty acid elongation and metabolism, and *N*-glycan biosynthesis (Table S3).

Taxonomic annotations of the differentially abundant KOs provided an overview of which taxa were responsible for the observed functional changes (Fig. 7C). For instance, the family Atheliaceae, which did not seem to be strongly affected by NE in the ITS and RNA data sets (Fig. 5A), accounted for a much larger proportion (25% versus 10%) of the lower-abundance than the higher-abundance KOs.

### Differential abundance analysis of the genus *Cortinarius* revealed extensive transcriptional downregulation.

To highlight the ability to extract insight into the functional response of specific taxa, all transcripts assigned to the genus *Cortinarius* were selected. *Cortinarius* is a widespread and common genus of ECM fungi that has been previously reported to be negatively affected by N enrichment in different contexts (28, 32, 33). In the current study, *Cortinarius* was still observed at low abundances after 25 years of treatment. We used a hierarchical clustering method (Ward minimum variance) and visualized the gene expression patterns of *Cortinarius* in our data set (Fig. 8A). This identified three main clusters of genes that had similar expression abundance patterns. Cluster 1 (4,047 transcripts) contained genes showing high abundance in the unperturbed control plots, which mostly dropped beyond detection after long term NE. Cluster 2 (1,456 transcripts) displayed high gene abundance in part of the ND and part of the NE samples, without any apparent pattern, while being mostly absent in other samples. Genes in cluster 3 (1,741 transcripts) displayed erratic abundance, with subsets of the cluster showing very high abundance in some samples, while other subsets displayed high abundances in other samples, with a tendency to be more abundant in control samples. Summarizing transcripts to KOs where possible, we observed substantial functional overlap between the three clusters (Fig. 8A, Venn diagram).

Gene Ontology (GO) enrichment of the three clusters identified significant enrichments for cluster 1 and cluster 3 (Fig. 8B). Cluster 1 showed significant (*P* < 0.001) enrichment in functions associated with the processing of organic macromolecules, including aromatic and heterocyclic compounds. Cluster 3 was enriched for a greater variety of GO terms, including metabolism of both small molecules and macromolecules, metabolism of organonitrogen compounds and organophosphates, and carbohydrate and carbohydrate derivative metabolism. Both cluster 1 and cluster 3 showed a reduction in abundance after long-term NE, but this effect was much more pronounced in cluster 1.

## DISCUSSION

RNA-Seq, in principle, enables studies of both the composition of active microbial communities and the biological functions being expressed by the constituent members. While several pipelines for the analysis of metatranscriptome data have been published (34–37), including evaluation of how the results from such data compare to those of amplicon- or whole-genome shotgun-based methods, these studies primarily focused on bacterial communities or relatively low-complexity species mixes or used the total RNA pool (21, 37, 38). Other studies have used both RNA-Seq and amplicon sequencing together to describe microbial communities from a taxonomic and functional perspective (18, 39). Here, we performed a study to ascertain how polyadenylated mRNA-Seq-based metatranscriptomics and DNA amplicon-based metagenomics results compare when profiling the complex root-associated and phyllospheric fungal communities associated with the boreal forest tree species Norway spruce (*Picea abies*) under natural and perturbed nutrient conditions. To facilitate the analysis of the metatranscriptomics data, we implemented a reproducible bioinformatic workflow to assemble fungal transcripts from the RNA-Seq data and subsequently annotate the assembled transcripts both functionally and taxonomically (Fig. 1). Creation of this custom workflow was necessitated by the lack of available tools for this specific case where our *a priori* criteria were the ability to (i) separate fungal and host reads and (ii) perform a *de novo* assembly of transcripts. We chose to assemble transcripts over direct read-based alignment to increase query sequence length and to maximize the number of aligned reads (Fig. S6) and because representative databases are lacking for our samples. Among previously published workflows, the IMP (Integrated Meta-omic Pipeline) workflow (37) supports host filtering and *de novo* assembly but was written with the human bacterial microbiome in mind and does not offer additional analyses such as differential expression or multiple sample comparisons (40). For the purpose of this study, we reanalyzed previously published ITS amplicon sequencing results from the same samples (28) to reflect new developments in sequence processing algorithms (41, 42).

10.1128/mSystems.00884-20.7 FIG S6 Read alignment statistics summary. Percentage of reads assigned to an open reading frame (ORF) with the three assemblers tested (megaHIT, Trans-ABySS, and Trinity) versus directly aligning reads to the reference database. Plots were split by treatment, and samples are colored by seasonal time point. Download FIG S6, EPS file, 0.5 MB. Copyright © 2021 Schneider et al. 2021 Schneider et al. https://creativecommons.org/licenses/by/4.0/ This content is distributed under the terms of the Creative Commons Attribution 4.0 International license.

Comparison of the control samples from roots and needles in the two data sets at the transcript and SOTU level revealed strikingly similar patterns of between-sample variation (Fig. 2), despite the proportionally higher number of needle SOTUs compared to the low number of fungal transcripts in needle samples. The low number of fungal transcripts obtained from the phyllospheric samples likely resulted from (i) the orders-of-magnitude-lower fungal load in the phyllospheric samples, leading to a much lower ratio of host to fungal nucleic acid in the extracts (43–45), and (ii) the higher phyllospheric fungal richness (Fig. 2) leading to sparser transcript count data in the needle samples. This low signal-to-noise ratio propagated through all further analyses of the needle samples, and for this reason we concentrated on the root data for in-depth analyses. This highlights the importance of relative fungal load for the application of this approach.

A further limitation of metatranscriptomics studies, especially where communities are composed of nonmodel systems, is the relatively low availability of sequenced reference fungal genomes. At the time the analyses were performed, there were 1,164 sequenced fungal genomes available at the JGI MycoCosm resource (46). This number is increasing steadily (1,681 at the time of writing) but is still far from being sufficiently comprehensive to capture a substantial portion of the fungal diversity present in most ecosystems. While the use of ITS amplicon data has its own limitations and methodological issues, it could be expected to yield a more comprehensive catalogue of taxa present in the sample due to the more established database resource currently available. Comparing the fungal metatranscriptomics assembly to the ITS data from the same samples, the taxonomic overlap was found to be small at lower taxonomic levels (Fig. 3). While only a relatively low proportion of transcripts and SOTUs were assigned at the species level (42 and 39%, respectively), this small overlap is likely to also stem from the already low overlap between the UNITE and the MycoCosm databases at low taxonomic levels, with only 40% of species present in the MycoCosm resource being represented in the UNITE database (Fig. 3A). This issue currently limits RNA and ITS comparability on taxonomic terms, but this is likely to improve as fungal genomes become increasingly available and as the UNITE database increases the number of included ITS sequences.

Furthermore, the two methods showed a consistent variation in evenness (Fig. 4C), and both identified a consistent decrease in the proportion of reads belonging to the 12 most abundant family annotations in response to nutrient enrichment (Fig. 5A). For both data sets, this appeared to reflect higher diversity and loss of dominance of certain groups of fungi, as reported previously for the ITS data set (28). That some families strongly differ in abundance between the two data sets might result from methodological bias in the ITS amplicon data (9), but it was shown previously that while most DNA and RNA data correlate fairly well, total gene expression abundances of some groups deviate in their levels from what could be expected when looking only at genomic DNA abundance (47). Moreover, it has been found that even ITS amplicons obtained from DNA and RNA in soil fungal communities yielded very different taxonomic compositions (38). Finally, a characteristic of mixed-species RNA-Seq is that transcript abundance captures both expression and species abundance; i.e., a higher abundance could stem from either higher gene expression per nucleus or a higher nucleus count. Current sequencing library generation protocols do not allow these two factors to be separated, although future strategies will likely overcome this, for example, through use of long-read sequencing technologies, such as Pacific Bioscience or Oxford Nanopore, that do not require assembly or via the use of unique molecular identifiers and transcript assembly algorithms that utilize this information.

While taxonomic overlap was low between the metatranscriptomics and ITS amplicon sequencing data, the congruence between unsupervised ordination methods was high, not only when comparing control samples but also when comparing the root sample clustering by nutrient status (Fig. 4A and B). The phyllospheric results are discussed in Text S1. As an additional approach to assess the similarities and differences between the two data types, we performed random forest classifications of NE-25 and ND samples in both data sets. We found that in a direct comparison, the random forest classification performed better on the ITS data, especially for needle samples. Congruent with the previous statistical tests, the random forest classifier found a strong effect of NE and no seasonal effect on root fungal communities. Another interesting observation was the notably higher correlation between mean relative abundance and importance in the ITS compared to RNA data. This could again be an artifact of the low taxonomic annotation in the RNA data, but potentially it indicates that the metatranscriptomic data enables a higher resolution by containing both taxonomic and expression abundance. Several previous studies have shown the importance of low abundance community members in a functional context (38, 48–50).

We applied random forest and differential abundance analyses to demonstrate use of the RNA-Seq data to provide both functional and taxonomic insights into the root-associated fungal community of Norway spruce and how it is affected by NE. Random forest classification accuracy when using KO counts, in comparison to taxonomic annotations at the species level, proved to be similarly good when classifying by treatment and slightly better when classifying by sampling date. The slightly higher accuracy for sampling date in both roots and needles when using functional profiles indicates that over the course of the season, shifts in functions expressed by fungal species are more pronounced, and yield higher signal strength, than the turnover of the species themselves. Both random forest and differential ortholog and transcript abundance analyses identified a number of functional categories with enrichment for transcripts having increased or decreased abundance as a result of the treatment, with highly congruent results from the two methods (Fig. 6 and 7). Furthermore, both methods were used to provide taxonomically resolved insights, identifying species and families that were important in explaining the separation either of the two treatment conditions (Fig. 5 and 7) or of transcripts from a specific family having significant changes in relative abundance (Fig. 7).

Finally, we demonstrated that we can pick one taxon of interest and investigate its specific transcriptomic response to the experimental conditions with the same methods, in this case, the known nitrophobic genus *Cortinarius* (Fig. 8). *Cortinarius* is one of the most species-rich ECM genera, with hundreds of species occurring in Sweden alone, and belongs to a group of ECM fungi that exhibit medium-distance fringe-type exploration and that have been shown in several studies to be sensitive to N addition (33). This N sensitivity has been hypothesized to be caused partly by their reliance on mobilizing organic N sources, using oxidative enzymes for degradation (32, 51). The high carbon cost of this foraging strategy would become disadvantageous with high inorganic N availability, due to both the decreased allocation of tree carbon belowground (30, 31) and a decrease in the energetical efficiency of oxidative enzymes (52). More recent comparative genomic studies have shown that Cortinarius glaucopus (the only sequenced European *Cortinarius* species to date) has retained an unusually high number of genes for plant cell wall-degrading enzymes in its genome, compared to most other ECM fungi (53). While the majority of *Cortinarius* transcripts in our data set showed a strong and uniform reduction in abundance after long-term NE, groups of genes had more varied, limited, or no response to the treatment (Fig. 8).

The large extent of functional overlap between the three identified gene clusters could suggest that each cluster represents a different *Cortinarius* species (or group of species), each of which has a different level of sensitivity to N addition. We observed an enrichment of GO terms associated with metabolic processing of aromatic compounds in the cluster of genes that showed the strongest consistent decrease in abundance after long-term NE (cluster 1), potentially indicating a strong reliance on the degradation of phenolic compounds (e.g., lignin derivatives) for this species, in line with the aforementioned literature. Cluster 2 (equal expression representation in both conditions) did not yield significant GO enrichments, which could suggest that some *Cortinarius* species are not as negatively affected by high N content. The third cluster showed variable representation in control samples and similarly variable (but overall reduced) representation after long-term NE. The higher number of enriched GO terms, including many important metabolic processes, such as carbohydrate and organonitrogen utilization, potentially indicates that this group of transcripts is from a *Cortinarius* species that relies on other enzymatic mechanisms to obtain N and that are not as dependent on tree derived C as the species in cluster 1. While these interpretations are, admittedly, speculative, they serve to highlight the additional power provided by a metatranscriptomic approach for enabling functionally informed insights and hypothesis generation to direct subsequent studies.

In conclusion, we have demonstrated that RNA-Seq metatranscriptomics, under the prerequisite of a sufficient microbial load in the sample of interest, has the potential to bypass the inherent limitations of ITS amplicon sequencing, especially with further technology development and availability of more extensive databases of sequenced genomes. We have shown that in terms of alpha and beta diversity, comparable results can be obtained using only metatranscriptomic data, while ITS amplicon data are still currently needed to provide a more complete taxonomic profile of the fungal community. While we did not consider this in the current study, the data generated additionally capture transcriptome dynamics in the host tree, enabling a plethora of additional analyses apart from what we demonstrated here. In conjunction with host tree expression, as well as other microbial communities, the presented data set and the approach in general hold great potential to yield insights into the dynamics of multispecies and multidomain gene expression and their interactions.

## MATERIALS AND METHODS

### Sample collection, nucleic acid extraction, and sequencing.

Samples were collected during the growing season in 2012 and stored at −80°C. The ITS1 amplicon sequencing data used in this study were published previously (28) and reanalyzed for this study. RNA was extracted from the same spruce root and needle samples in early 2013 and used for RNA sequencing; the details are described in Text S1. RNA was successfully sequenced from 214 samples, 107 root and 107 needle samples. For ideal comparability to the ITS data, replicates from within one on-site block were pooled, resulting in 36 pooled root and needle samples.

### Metatranscriptomic workflow.

Preprocessing and analysis of metatranscriptomic data were implemented in a Snakemake workflow available on Bitbucket (54); we ensured complete, hardware-agnostic reproducibility through implementation in both docker and singularity containers. A detailed description of software and parameters used in the workflow is available in Text S1. Briefly, raw reads were trimmed and filtered using cutadapt and Trimmomatic (55, 56). Read quality scores before and after preprocessing were assessed with FastQC and MultiQC (57, 58). Fungal reads were selected from preprocessed reads aligned using bowtie2 against the JGI MycoCosm database (46, 59) and TaxMapper against its own database (60). Reads aligning to JGI MycoCosm were filtered for host reads by bowtie2 alignments against the Norway spruce reference genome obtained from PlantGenIE (61). We deduplicated fungal read pairs using FastUniq (62). Subsequently, fungal reads were assembled using Megahit, Trans-ABySS, and Trinity (63–65). Open reading frames (ORFs) in the Megahit assembly were identified using GeneMarkS-T (66). FeatureCounts from the subread package (67) was used to count the reads aligning within ORFs with bowtie2. Raw counts were normalized to transcripts per million (TPM) (68). Translated protein sequences were annotated using eggnog-mapper in conjunction with the eggnog database (69, 70), to obtain KEGG ortholog (KO) and Gene Ontology (GO) annotations (71). For taxonomic annotation, we used a database comprising proteins constructed from JGI MycoCosm, TaxMapper, and the Hygrophorus russula MG78 genome with genes predicted using Augustus (72). *Hygrophorus russula* was included to account for the high abundance of *Hygrophorus* at the field site, observed both in the ITS data and *in situ* sporocarp assessments. Taxonomy was assigned to transcripts using contigtax (73), which uses rank-specific thresholds (74) to infer lowest common ancestors, based on DIAMOND BLASTX searches (75).

### Amplicon sequence data pipeline.

The code needed to run the preprocessing and analysis of the amplicon sequencing data is available on GitHub (76). A detailed description is available in Text S1. In short, raw reads were demultiplexed using deML (77), and primer sequences were removed using cutadapt (55) after pooling technical replicates, as described previously (28). The R package dada2 (42) was used to filter and denoise the reads, before dereplicating them into amplicon sequencing variants (ASVs), merging overlapping forward and reverse reads, and removing chimeric sequences. The ITS1 region was cut out from the ASVs using ITSx (78) and subsequently clustered into Swarm operational taxonomic units (SOTUs) using Swarm (41). Taxonomy was assigned using naive Bayesian classifier implemented in dada2, with the UNITE database as a reference (79).

### Analyses and visualizations.

All further analyses were performed using R (80), unless otherwise specified. Visualizations were plotted using ggplot2, unless otherwise specified (81). Venn diagrams in Fig. 2 were created using the R package VennDiagram (82), and Venn diagrams and correlations in Fig. 3 were created using jupyter and matplotlib (83, 84). Detailed parameter information can be found in the above git repositories, and a more detailed description is provided in Text S1. Amplicon sequencing data were filtered and rarefied using vegan (85), which was also used for PERMANOVA, Shannon diversity, and Mantel and Procrustes tests. The package phyloseq (v 1.28) was used to visualize PCoA ordinations (86). Linear mixed-effect models were used to test for significant differences in diversity using the nlme package (87) and the multcomp package (88). RNA-Seq-derived transcripts were selected to be of fungal origin and subsequently filtered using the same criteria as for the SOTUs. After filtering, the replicates per plot were merged by mean value to make the data more comparable to the ITS amplicon data. Filtered metatranscriptome count data were transformed using the function varianceStabilizingTransformation from the DESeq2 package prior to principal-component analysis (89). Random forest analyses were implemented using the RandomForestClassifier from scikit-learn (90). Heat maps summarizing random forest results were plotted using matplotlib (84), while the heat map in Fig. 8 was plotted using the R package pheatmap (91). DESeq2 was used to identify differentially abundant KOs and transcripts (89). Differentially abundant KOs and transcripts were filtered to have a log fold change of at least 0.5 and a *P* value of <0.05. Functions for easier filtering and visualization of differential expression results were pulled from the Rtoolbox repository (92). The tool gofer2 (93) was used for KO and Gene Ontology enrichment, the R wrapper of which was pulled from the repository of the Umeå Plant Science Centre bioinformatics facility (94). The R package treemap was used to visualize the enrichments (95).

### Data availability.

The raw data from the ITS1 amplicon sequencing have been deposited in the European Nucleotide Archive (ENA) with accession number PRJEB21692 (96). RNA-Seq raw data are also deposited in the ENA with accession number PRJEB35783 (97). Workflows and scripts to preprocess and analyze the data have been made available in the git repositories mentioned above.
