# Supplementary material for: Comparative Fungal Community Analyses Using Metatranscriptomics and Internal Transcribed Spacer Amplicon Sequencing from Norway Spruce
Source: mSystems. 2021 Feb 16;6(1):e00884-20. doi: 10.1128/mSystems.00884-20 (PMC8573963; doi:10.1128/mSystems.00884-20)
Supplement: TEXT S1 [file msystems.00884-20-s0001.docx]

**Text S1**

Supplementary methods

Sample collection, nucleic acid extraction, and sequencing

The ITS1 amplicon sequencing data used in this study were published previously (1), and re-analyzed for this study. Samples were collected during the growing season in 2012 and stored at -80 °C. In January 2013, RNA was extracted from the same spruce root and needle samples used for the above ITS1 amplicon analysis. Roots and needles were ground manually in liquid nitrogen using a mortar and pestle. Total RNA was isolated from 75 mg of tissue using the Plant RNA Isolation Aid and the RNAqueous kit (Invitrogen: Waltham, Massachusetts, USA), followed by a DNA-free kit (Invitrogen) DNase treatment. LiCl precipitation followed by a 70% ethanol washing step was performed to ensure a better quality of RNA and to deplete short RNAs. For samples from the ND plots, two or three samples were pooled before LiCl precipitation to reach the required amount of RNA. Nucleotide concentration was determined using a Nanodrop ND100 (NanoDrop Technologies, Delaware, USA) and RNA quality was assessed using a Bioanalyzer (Agilent 2100 Bioanalyzer, Agilent Technologies, Waldbronn, Germany).

The RNA was prepared for sequencing using Illumina TruSeq Stranded mRNA followed by polyA selection and the RNA sequencing was performed on a HiSeq2500, yielding 101bp paired-end reads, both at the Science for Life laboratory (Stockholm, Sweden). RNA was successfully sequenced from 214 samples, 107 root and 107 needle samples. Needle and root samples were sequenced in 2014 and 2017, respectively. For ideal comparability to the ITS data, replicates from within one on-site block were pooled, resulting in 36 pooled root and needle samples, respectively.

Metatranscriptomic workflow

Preprocessing and analysis of metatranscriptomic data was implemented in a snakemake workflow available on Bitbucket (2), we ensured complete, hardware agnostic reproducibility through implementation in both docker and singularity containers.

**Preprocessing and read filtering**

Adapters were removed using cutadapt (v. 1.18) with error-rate set to 0.2 (-e 0.2), followed by quality trimming with Trimmomatic (v. 0.38) with settings ‘LEADING:25 TRAILING:25 SLIDINGWINDOW:4:15 HEADCROP:2 MINLEN:50’ (3, 4). Read quality after preprocessing was investigated using fastqc (v. 0.11.7) and MultiQC (v. 1.5) (5, 6).

Preprocessed reads were filtered to obtain a fungal-specific dataset for downstream analyses. First, reads were aligned against a set of 16,348,745 fungal transcripts downloaded from JGI Mycocosm (7) using bowtie2 (v. 2.3.4.3) with settings ‘--very-sensitive’ and read-pairs with both ends aligned were kept (8). Read statistics are shown in Table S1. Second, the kept reads were aligned again with bowtie2 against 66,632 Norway spruce transcripts downloaded from PlantGenIE and read-pairs with both ends aligned were discarded (9). In parallell, reads were classified using taxmapper (v. 1.0.2) and read-pairs classified as Fungi were kept (10). Finally, the union of reads from bowtie2 and taxmapper filtering was used in downstream analyses.

**Assembly**

Fungal read-pairs were pooled and deduplicated using FastUniq (v. 1.1) (11). Deduplicated reads were assembled using Megahit (v. 1.1.3) with settings ‘--prune-level 3 --min-contig-len 200’ (12).

Assemblies were also generated using Trans-ABySS (v. 2.0.1) and Trinity (v. 2.9.0) on the deduplicated reads (13, 14). Trans-ABySS was run with kmer sizes 21, 29, 39, 59, 79, 99 and 119 and minimum contig length of 200 followed by merging the assemblies with transabyss-merge. Trinity was run using a singularity image (singularity v. 3.5.0-1.el7) with default settings.

**Annotation**

Open reading frames (ORFs) were identified on the assembled transcripts using GeneMarkS-T (v.  5.1) with default settings (15). Preprocessed and fungal-filtered (but non-deduplicated) read-pairs were aligned to the transcripts using bowtie2 (version and settings as above) and reads aligned within open reading frames were counted using FeatureCounts from the subread package (v. 1.6.2) with settings ‘-p -B -Q 10’ to count paired-end reads with both ends aligned and a quality score of at least 10 (16). Raw counts were normalized to transcripts per million (TPM) as described in (17).

Translated protein sequences were annotated using eggnog-mapper (v. 2.0.1) and version 5.0 of the eggNOG database (18, 19). Functional annotations were quantified by summing the raw and normalized read counts from ORFs to the respective annotations.

**Taxonomic annotation**

In order to assign taxonomy to transcripts we compiled a custom database of protein sequences from 1 164 fungal genomes downloaded from the JGI Mycocosm resource as well as 121 genomes from the taxmapper database (of which six were fungal). In addition, the *Hygrophorus russula* MG78 genome was downloaded from NCBI and genes were predicted using Augustus (v. 3.2.3) with the laccaria_bicolor model (20). *Hygrophorus russula* was added to account for high abundance of the genus *Hygrophorus* at the site, determined both by the ITS amplicon data analysis, and *in situ* sporocarp assessments. The final protein database consisted of a total of 17 694 143 protein sequences (14 976 193 from JGI, 2 708 401 from taxmapper and 9 549 from *H. russula*). Taxonomy was assigned to the assembled contigs using the tool contigtax (v. 0.5.7) (21) and this custom database. Contigtax uses diamond (v. 0.9.22) to query contigs in a translated ‘blastx’ search, keeping hits for each query with an e-value<0.001 and a bitscore within 5% of the top alignment score (22). From this set of hits contigtax attempts to assign the most resolved taxonomy in the NCBI taxonomy tree starting at species, followed by genus and phylum only considering hits with at least 85%, 60% or 45% identity at each rank, respectively, according to (23). If the taxonomy of all considered hits at a taxonomic rank is congruent, that taxonomy is assigned and higher ranks inferred from the NCBI taxonomy tree while prefixing the assigned taxonomy with ‘Unclassified’ for lower ranks (if any).

Amplicon sequence data pipeline

The code needed to run the preprocessing and analysis of the amplicon sequencing has been made available on github (24).

**Preprocessing, denoising and ASV clustering**

The raw ITS reads were demultiplexed into files per sample using deML (Release 10.06.2016) (25). This includes the needle and root samples needed for the analysis as well as mock community samples, with the same composition as described previously (1). Replicates from the same block were merged before processing, in line with the previous publication using this data (1). Reads were cut to the desired fragment using the PCR primer sequences and cutadapt (v. 2.4) (3). The subsequent steps were performed using dada2 (v. 1.14) (26). Needle and root samples were preprocessed separately. The cut reads were filtered and trimmed using the parameters ’maxN = 0, maxEE = c(6,6), truncQ = 2, minLen = 50, rm.phix = TRUE’. Filtered reads were then used to learn errors, before dereplicating, denoising, and finally merging the forward and reverse denoised reads with a maximum allowed mismatch of 1 base. Chimeras were removed using the “consensus” method. The resulting set of amplicon sequence variants (ASVs) was used for further processing and clustering.

**Extraction of ITS region and Swarm clustering**

The selection and excision of the target ITS1 region was done using ITSx (v. 1.1.2), also allowing for partial ITS sequences and requiring a minimum length of 50 basepairs (27). The resulting excised ITS1 region sequences were once again dereplicated. At this point needle and root sample sets were combined for final clustering. For every ASV, the counts from all samples were combined and output to a fasta file. Swarm (v. 2.2.2) was used to cluster these ASVs into SOTUs (Swarm operational taxonomic units) (28). The clustering was optimized to using the parameter “d -3” by comparing the theoretical mock community to the one obtained with different clustering settings. Counts were summarised to the obtained clusters, and taxonomy was assigned to all SOTUs using the naive Bayesian classifier, as implemented in dada2. UNITE (v. 8.0; 2018-11-18) was used as a fungal reference database (29).

Further analyses and visualizations

All further analyses were performed using R (v 3.6.2) (30), unless specified otherwise. Visualizations were plotted using ggplot2, unless mentioned (31). Venn diagrams in Figure 2 were created using the package VennDiagram (v 1.6.20) (32) and venn diagrams and correlations in Figure 3 were created using jupyter and matplotlib (33, 34). Detailed parameter information can be found in the git repositories indicated above.

**Amplicon sequencing data**

SOTUs were required to have at least five reads in at least two replicates of a sample type, as well as at least 0.005% total abundance in any sample type. Before running principal coordinate analyses (PCoA), samples were rarefied to minimum sequencing depth using vegan (v 2.5.6) (35). The package phyloseq (v 1.28) was used to visualize the ordinations (36). Communities were tested for significant differences between treatments and timepoints using permutational multivariate analysis (PERMANOVA), implemented in the adonis function from the vegan R package (35). Shannon diversities were calculated using vegan. Linear mixed-effect models (Diversity~Treatment) were used to test for significant differences in diversity using the lme function from the nlme package (v 3.1) (37). In case of significance (p<0.05), Tukey honestly significant differences (HSD) were calculated using the function glht from the multcomp (v 1.4) R package (38).

**Metatranscriptome data**

Transcripts were filtered to be of fungal origin and subsequently filtered by abundance using the same criteria as the SOTUs. After filtering, the replicates per plot were merged by mean value to make the data more comparable to the ITS amplicon data. Filtered metatranscriptome count data was transformed using the function varianceStabilizingTransformation from the DESeq2 package (v 1.24.0) prior to principal component analysis (39).

All instances of mantel and procrustes tests to compare amplicon and transcript ordinations and distances were performed using vegan (v 2.5.6) (35).

**Random forest analyses**

Random forest analyses were implemented using the RandomForestClassifier from scikit-learn (v. 0.20.2) with settings ‘max_depth=None, min_samples_split=2, max_features=”sqrt”, oob_score=True’ and using 25% of samples as a training set (40). To assess accuracy for the various datasets models were fitted with the number of trees set at 250, 500, 750 and 1000 and repeated with three different random state values (0, 1, 2). Feature importance was obtained from model fit using 1000 trees. The macro average recall and precision values were reported. Random forest analyses were performed on the replicate-merged dataset (see previous section), as well as the full dataset with all separate replicates, and without low abundance filtering applied. Heatmaps visualizing random forest results were plotted using matplotlib (34).

**Functional analyses**

DESeq2 was used to identify differentially abundant KEGG orthologs and transcripts between the long term NE condition and the ND control samples for every sampled seasonal timepoint (39). Differentially abundant KOs and transcripts were filtered to have a log fold change of at least 0.5 and a p-value <0.05 before further analyses. Functions for easier filtering and visualization of differential expression results were sourced from the ‘Rtoolbox’ repository (41). The tool gofer2 (42) was used for KO and gene ontology enrichment, the R wrapper of said tool was pulled from the public repository of the Umeå Plant Science Centre bioinformatics facility (43). The R package treemap was used to visualize the enrichments (44). The heatmap in Fig 8 was plotted using the R package pheatmap (v. 1.0.12), with clustering method “ward.D” and scaling by rows enabled (45).

Supplementary results and discussion

Pipeline development statistics

RNA-Sequencing of roots and needles yielded 15.6 Million (sd +/- 4 804 448) paired-end reads on average, of which 90-95%, 14.7 Million reads on average, remained after adapter/quality trimming (Fig 1A). On average, 0.6% (89 763 reads) and 6.7% (933 229 reads) were determined as fungal (by alignment to the JGI Mycocosm and taxmapper databases) in the needle and root samples, respectively (Table S1). Megahit assembly of fungal reads generated 615 331 transcripts, with a total size of 444 Mbp. Using alternative assemblers (Trans-AbySS and Trinity) resulted in larger total assembly size, but lower length statistics and a reduced number of reads assigned to open reading frames (ORFs) called on transcripts (Fig S1). The longest transcript spanned 12 588, and the N50 length of the assembly was 822 bp (For a ExN50 graph, see Fig S1). 547 305 ORFs were called on the assembled transcripts, with a median length of 98 amino acids. For 68 029 (11.1%) transcripts no ORF was found. Reads were mapped back to the ORFs, with an average of 34.3% assigned reads in needle samples and 70.6% in root samples. Functional annotation of ORFs was performed, with 92.7% of ORFs having a hit in the eggnog database, of which 59.8% were assigned to a Kyoto Encyclopedia of Genes and Genomes (KEGG) Ortholog. Taxonomic assignments resulted in 95.5%, 50.4% and 34.2% of transcripts assigned at phylum, genus and species level, respectively.

For the ITS1 amplicon sequencing data between 86 279 and 338 800 reads  per sample remained after filtering (176 734 on average), corresponding to a range between 47 and 78% of the raw reads (Table S1). Denoising and chimera removal resulted in 2694 ASVs in root samples and 3032 in needle samples. After clustering, we obtained a total of 2673 SOTUs, 1172 in root samples and 1890 in needle samples of which 389 were shared between tree tissues.

Comparison of taxonomic annotations in ITS and RNA databases and datasets

To compare the coverage of the databases used for taxonomic annotation of transcripts (JGI Mycocosm and taxmapper) and SOTUs (UNITE database) respectively, the number of families, genera and species listed in both or only one of the databases was assessed (Fig 3a). The proportional overlap between the two databases clearly decreased with lower taxonomic levels. A similar trend was found for taxa identified in the two datasets, but with a lower proportional overlap at the species level than between the databases (Fig 3A, lower row). At the family level, the percentage of common transcripts and SOTUs was around 50%, while at the species level <5% remained for the overlap of both datasets. The same trend was apparent for the proportion of reads mapped to transcripts/SOTUs: in root samples 50-75% of reads were mapped to common taxa at family and genus level with only 0-5% at the species level (Fig 3A, Fig S2A).

To assess how well the relative abundance of common taxa agreed between the RNA and the ITS datasets, spearman rank correlations were computed for samples at family, genus, and species level (Fig 3B). At the family level, the correlations ranged between 0.4 and 0.6 (median 0.53 in roots and 0.48 in needles). Correlations decreased rapidly at lower taxonomic ranks in needle samples (median 0.48 and 0.26 at genus and species, respectively), and moderately in root samples (median 0.53 and 0.44 at genus and species, respectively).

Comparison of ordinations and Shannon diversity index in needle samples

Needle samples showed a strong seasonal effect (p<0.001) in both datasets, and a highly significant treatment effect in the ITS data (p<0.001). The treatment effect was only moderately significant for the fungal transcripts in the needle samples (p=0.03). The ordinations were visually dissimilar and only significantly correlated according to the procrustes test, and with only a weak to moderate correlation coefficient (Fig S3A, Fig S3B; Mantel r: 0.058, p=0.26; Procrustes correlation 0.31, p=0.006).

In the needle samples the shannon diversity index correlation was weak (Fig S3C, Pearson r = 0.10). The treatment had no significant influence on Shannon diversity in either dataset. Looking at the effect of the sampling timepoint on Shannon diversity in both datasets, we observed a highly significant (p<0.001) difference between the second timepoint (late June) and the first (early June) and last (October) timepoints in the ITS dataset, as well as a weaker difference (p=0.009) between samples taken in August and late June.

Comparison of random forest classification on species level - detailed results

We used a random forest classifier on taxonomic (RNA and ITS) profiles to classify samples by treatment and date and to identify features (taxonomic units) of highest importance in explaining sample classifications. To compare random forest performance with maximum number of replicates and gene counts, the RNA analysis was also run without averaging the replicates per plot and without filtering of low abundance transcripts, henceforth referred to as the “full dataset” in contrast to the averaged dataset used for all other analyses, on which abundance filtering and replicate averaging were applied.

There was high predictive accuracy of treatment type (‘Control’, ‘5 year’ and ‘25 year’) for root samples at the family, genus and species level (median accuracy 0.89-1.0 and 0.67-0.78 for ITS and RNA respectively) (Fig S4A). In needle samples treatment prediction accuracy was high for the ITS data at the family-species level (0.94 - 1.0 median accuracy) but low for the RNA data (0.33 median accuracy). A binary classifier run on Control/25 year samples resulted in an accuracy of 1.0 at family to species levels in ITS roots and needles. For the RNA data the binary classifier had median accuracy of 1.0 at the family-species level in root samples and 0.83 – 1.0 median accuracy in needle samples.

Prediction accuracy for sampling date was higher in needles compared to roots at family to species level in both ITS (0.56 – 0.67 vs. 0.11-0.22) and RNA data (0.89 vs. 0.22) (Fig S4B). Simultaneously predicting treatment and sampling date resulted in low accuracy overall for both methods (data not shown). Summarized statistics of model accuracy are shown in Table S2. Using all replicates as well as all species in the RNA dataset resulted in higher prediction accuracy for treatment in both roots and needles (0.93 – 0.96) (Fig S4C). Model accuracy for time of sampling was also higher in roots in the full dataset (0.65 – 0.72). A binary classifier for Control and 25 year resulted in similar accuracy for full and averaged datasets.

We then investigated which species were most important when classifying root samples belonging to ‘Control’ and ‘25 year’ groups. In ITS data, the 30 most important species had a summed importance of 0.69 and belonged to families including Cortinariaceae, Atheliaceae, Thelephoraceae, Myxotrichaceae and Mortierellaceae. These families, with the exception of Mortierellaceae, were among the 12 most abundant in the ITS dataset. In addition, the mean relative abundance of a species and its feature importance had a spearman rank correlation coefficient of 0.79. For the averaged RNA data, the top 30 most important species had summed importance of 0.4 and belonged to families including Strophariaceae, Pisolithaceae, Cortinariaceae and Thelephoraceae. In contrast to the ITS dataset, most of the top 30 important species in the RNA dataset did not belong to the most abundant families and the spearman correlation for mean relative abundance and importance was only 0.13. In both the ITS and RNA datasets, clustering samples using the abundance of the top 30 most important species resulted in complete separation of control and 25 year samples (Fig. 5B and 5C). Species belonging to genus *Pisolithus* were differentially abundant in control and 25 year samples with *Pisolithus croceorrhizus* and unclassified members of *Pisolithus* more abundant in control samples and *Pisolithus sp.* (corresponding to *Pisolithus thermaeus* in our database) being more abundant in 25 year samples (Fig. 5C). A similar pattern was seen when using the full RNA dataset (Fig S5).

Discussion – phyllospheric samples

In the phyllospheric samples, both seasonal and treatment effects were observed in the two datasets (Fig S3), leading to non-congruent ordinations due to the difference in strength of season and treatment (treatment stronger in ITS, season stronger in RNA) effects on taxonomic composition and transcript expression dynamics. The higher correlation of statistical comparisons in the roots than in the needles suggests either that the phyllospheric transcriptome was not captured sufficiently compared to the roots, as discussed in the main text, or that there was actually a greater deviation of gene expression and taxonomic composition in the phyllospheric fungal community. The stronger seasonal signal in RNA data suggests that expression profiles of species change more extensively than species composition, as supported by the random forest analysis (see below).

As an additional approach to assess the similarities and differences between the two data types, we performed random forest classifications of 25 year nutrient enrichment and control samples in both datasets. We found that in direct comparison the random forest performed better on the ITS data, especially in needle samples. This is likely the result of a stronger signal to noise ratio obtained in needle samples with targeted amplicon sequencing, the low accuracy in the RNA data resulting from the very low microbial load and resulting representation of fungal reads, as discussed in the main text. Prediction accuracy for sampling date indicated a clear seasonal profile in the fungal community in needle samples, with low seasonal signal in the root samples, indicative of a more stable community in roots in terms of taxonomic and functional composition. In contrast, there was a more pronounced influence of nutrient enrichment in roots compared to needles. This matches very well with the statistical results discussed earlier. Finally, we also saw that we could increase classification accuracy in the RNA data by not summarizing block replicates and not filtering low abundant transcripts, indicating that the metatranscriptomic data has potential for even higher sensitivity in random forest analyses by fine-tuning filtering and summarization steps.

References

1.    Haas JC, Street NR, Sjödin A, Lee NM, Högberg MN, Näsholm T, Hurry V. 2018. Microbial community response to growing season and plant nutrient optimisation in a boreal Norway spruce forest. Soil Biol Biochem 125:197–209.

2.    Sundh J. 2020. N_Street_1801. Bitbucket <https://bitbucket.org/scilifelab-lts/n_street_1801/src/master/>. Retrieved 25 June 2020. {*Code and software.*}

3.    Martin M. 2011. Cutadapt removes adapter sequences from high-throughput sequencing reads. EMBnet.journal 17:10.

4.    Bolger AM, Lohse M, Usadel B. 2014. Trimmomatic: a flexible trimmer for Illumina sequence data. Bioinformatics 30:2114–2120.

5.    Andrews S, Krueger F, Seconds-Pichon A, Biggins F, Wingett S. 2015. FastQC. A quality control tool for high throughput sequence data. Babraham Bioinformatics. Babraham Inst.

6.    Ewels P, Magnusson M, Lundin S, Käller M. 2016. MultiQC: Summarize analysis results for multiple tools and samples in a single report. Bioinformatics 32:btw354.

7.    Grigoriev I V., Nikitin R, Haridas S, Kuo A, Ohm R, Otillar R, Riley R, Salamov A, Zhao X, Korzeniewski F, Smirnova T, Nordberg H, Dubchak I, Shabalov I. 2014. MycoCosm portal: gearing up for 1000 fungal genomes. Nucleic Acids Res 42:D699–D704.

8.    Langmead B, Salzberg SL. 2012. Fast gapped-read alignment with Bowtie 2. Nat Methods 9:357–359.

9.    Sundell D, Mannapperuma C, Netotea S, Delhomme N, Lin Y-C, Sjödin A, Van de Peer Y, Jansson S, Hvidsten TR, Street NR. 2015. The Plant Genome Integrative Explorer Resource: PlantGenIE.org. New Phytol 208:1149–1156.

10.  Beisser D, Graupner N, Grossmann L, Timm H, Boenigk J, Rahmann S. 2017. TaxMapper: an analysis tool, reference database and workflow for metatranscriptome analysis of eukaryotic microorganisms. BMC Genomics 18:787.

11.  Xu H, Luo X, Qian J, Pang X, Song J, Qian G, Chen J, Chen S. 2012. FastUniq: a fast de novo duplicates removal tool for paired short reads. PLoS One 7:e52249.

12.  Li D, Liu C-M, Luo R, Sadakane K, Lam T-W. 2015. MEGAHIT: an ultra-fast single-node solution for large and complex metagenomics assembly via succinct de Bruijn graph. Bioinformatics 31:1674–1676.

13.  Robertson G, Schein J, Chiu R, Corbett R, Field M, Jackman SD, Mungall K, Lee S, Okada HM, Qian JQ, Griffith M, Raymond A, Thiessen N, Cezard T, Butterfield YS, Newsome R, Chan SK, She R, Varhol R, Kamoh B, Prabhu A-L, Tam A, Zhao Y, Moore RA, Hirst M, Marra MA, Jones SJM, Hoodless PA, Birol I. 2010. De novo assembly and analysis of RNA-seq data. Nat Methods 7:909–912.

14.  Grabherr MG, Haas BJ, Yassour M, Levin JZ, Thompson DA, Amit I, Adiconis X, Fan L, Raychowdhury R, Zeng Q, Chen Z, Mauceli E, Hacohen N, Gnirke A, Rhind N, di Palma F, Birren BW, Nusbaum C, Lindblad-Toh K, Friedman N, Regev A. 2011. Full-length transcriptome assembly from RNA-Seq data without a reference genome. Nat Biotechnol 29:644–652.

15.  Tang S, Lomsadze A, Borodovsky M. 2015. Identification of protein coding regions in RNA transcripts. Nucleic Acids Res 43:e78.

16.  Liao Y, Smyth GK, Shi W. 2014. featureCounts: an efficient general purpose program for assigning sequence reads to genomic features. Bioinformatics 30:923–930.

17.  Wagner GP, Kin K, Lynch VJ. 2012. Measurement of mRNA abundance using RNA-seq data: RPKM measure is inconsistent among samples. Theory Biosci 131:281–285.

18.  Huerta-Cepas J, Szklarczyk D, Heller D, Hernández-Plaza A, Forslund SK, Cook H, Mende DR, Letunic I, Rattei T, Jensen LJ, von Mering C, Bork P. 2019. eggNOG 5.0: a hierarchical, functionally and phylogenetically annotated orthology resource based on 5090 organisms and 2502 viruses. Nucleic Acids Res 47:D309–D314.

19.  Huerta-Cepas J, Forslund K, Coelho LP, Szklarczyk D, Jensen LJ, von Mering C, Bork P. 2017. Fast Genome-Wide Functional Annotation through Orthology Assignment by eggNOG-Mapper. Mol Biol Evol 34:2115–2122.

20.  Stanke M, Keller O, Gunduz I, Hayes A, Waack S, Morgenstern B. 2006. AUGUSTUS: ab initio prediction of alternative transcripts. Nucleic Acids Res 34:W435–W439.

21.  Sundh J. 2020. contigtax. Github <https://github.com/NBISweden/contigtax>. Retrieved 29 April 2020. {*Code and software.*}

22.  Buchfink B, Xie C, Huson DH. 2015. Fast and sensitive protein alignment using DIAMOND. Nat Methods 12:59–60.

23.  Luo C, Rodriguez-R LM, Konstantinidis KT. MyTaxa: an advanced taxonomic classifier for genomic and metagenomic sequences https://doi.org/10.1093/nar/gku169.

24.  Schneider AN. 2020. its_workflow. Github <https://github.com/andnischneider/its_workflow>. Retrieved 15 December 2020. {*Code and software.*}

25.  Renaud G, Stenzel U, Maricic T, Wiebe V, Kelso J. 2015. deML: robust demultiplexing of Illumina sequences using a likelihood-based approach. Bioinformatics 31:770–772.

26.  Callahan BJ, McMurdie PJ, Rosen MJ, Han AW, Johnson AJA, Holmes SP. 2016. DADA2: High-resolution sample inference from Illumina amplicon data. Nat Methods 13:581–583.

27.  Bengtsson-Palme J, Ryberg M, Hartmann M, Branco S, Wang Z, Godhe A, De Wit P, Sánchez-García M, Ebersberger I, de Sousa F, Amend AS, Jumpponen A, Unterseher M, Kristiansson E, Abarenkov K, Bertrand YJK, Sanli K, Eriksson KM, Vik U, Veldre V, Nilsson RH. 2013. Improved software detection and extraction of ITS1 and ITS2 from ribosomal ITS sequences of fungi and other eukaryotes for analysis of environmental sequencing data. Methods Ecol Evol n/a-n/a.

28.  Mahé F, Rognes T, Quince C, De Vargas C, Dunthorn M. 2015. Swarm v2: highly-scalable and high-resolution amplicon clustering. PeerJ 3:e1420.

29.  Kõljalg U, Larsson KH, Abarenkov K, Nilsson RH, Alexander IJ, Eberhardt U, Erland S, Høiland K, Kjøller R, Larsson E, Pennanen T, Sen R, Taylor AFS, Tedersoo L, Vrålstad T, Ursing BM. 2005. UNITE: A database providing web-based methods for the molecular identification of ectomycorrhizal fungi. New Phytol 166:1063–1068.

30.  R Core Team. 2019. R: A Language and Environment for Statistical Computing. Vienna, Austria.

31.  Wickham H. 2009. ggplot2: elegant graphics for data analysis. Springer New York.

32.  Chen H, Boutros PC. 2011. VennDiagram: a package for the generation of highly-customizable Venn and Euler diagrams in R. BMC Bioinformatics 12:35.

33.  Kluyver T, Ragan-kelley B, Pérez F, Granger B, Bussonnier M, Frederic J, Kelley K, Hamrick J, Grout J, Corlay S, Ivanov P, Avila D, Abdalla S, Willing C, Development Team J. 2016. Jupyter Notebooks—a publishing format for reproducible computational workflows, p. 87–90. *In* Loizides, F, Schmidt, B (eds.), Positioning and Power in Academic Publishing: Players, Agents and Agendas. IOS Press.

34.  Hunter JD. 2007. Matplotlib: A 2D graphics environment. Comput Sci Eng 9:99–104.

35.  Dixon P. 2003. VEGAN, a package of R functions for community ecology. J Veg Sci 14:927–930.

36.  McMurdie PJ, Holmes S. 2013. phyloseq: An R Package for Reproducible Interactive Analysis and Graphics of Microbiome Census Data. PLoS One 8:e61217.

37.  Pinheiro J, Bates D, DebRoy S, Sarkar D, R Core Team. 2020. {nlme}: Linear and Nonlinear Mixed Effects Models.

38.  Hothorn T, Bretz F, Westfall P. 2008. Simultaneous Inference in General Parametric Models. Biometrical J 50:346–363.

39.  Love MI, Huber W, Anders S. 2014. Moderated estimation of fold change and dispersion for RNA-seq data with DESeq2. Genome Biol 15:550.

40.  Pedregosa F, Varoquaux G, Gramfort A, Michel V, Thirion B, Grisel O, Blondel M, Prettenhofer P, Weiss R, Dubourg V, Vanderplas J, Passos A, Cournapeau D, Brucher M, Perrot M, Duchesnay É. 2011. Scikit-learn: Machine Learning in Python. J Mach Learn Res 12:2825–2830.

41.  Serrano A. 2020. R toolbox. Github <https://github.com/loalon/Rtoolbox>. Retrieved 15 August 2020. {*Code and software.*}

42.  Schiffthaler B. 2018. Gofer 2. Github <https://github.com/bschiffthaler/gofer2>. Retrieved 25 June 2020. {*Code and software.*}

43.  UPSCb. 2020. UPSCb-common. Github <https://github.com/UPSCb/UPSCb-common>. Retrieved 15 August 2020. {*Code and software.*}

44.  Tennekes M, Ellis P. 2017. Package ‘treemap.’ R.

45.  Kolde, R. (2012). Pheatmap: pretty heatmaps. *R package*, *1*(2).
